# Glide Time Relates to Mediolateral Plantar Pressure Distribution Rather Than Ski Edging in Ski Skating

**DOI:** 10.3389/fspor.2020.00117

**Published:** 2020-09-18

**Authors:** Sébastien Pavailler, Frédérique Hintzy, Guillaume Y. Millet, Nicolas Horvais, Pierre Samozino

**Affiliations:** ^1^Sport Sciences Laboratory, Footwear Department, Salomon SAS, 14 Chemin des Croiselets, Épagny Metz-Tessy, France; ^2^Univ Savoie Mont Blanc, Inter-University Laboratory of Human Movement Biology, Chambéry, France; ^3^Univ Lyon, UJM-Saint-Etienne, Inter-University Laboratory of Human Movement Biology, Saint-Etienne, France; ^4^Institut Universitaire de France (IUF), Paris, France

**Keywords:** cross-country skiing, performance, asymmetry index, inertial measurement unit, submaximal speed, V2 technique

## Abstract

The purpose of this study was (i) to assess the differences in relative glide time and both ski edging angle and plantar pressure mediolateral distribution in skiers of different levels and (ii) to further investigate the relationships between the aforementioned variables. Twelve male cross-country skiers (6 national and 6 regional level) skied at 4.2 m s^−1^ on a 2.5° uphill snow track using the V2 technique. The relative glide time (in percentage of contact time) and mediolateral plantar pressure distribution variables (asymmetry index, ASI) were derived from pressure insole measurements. Ski edging angle variables were calculated from an Inertial Measurement Unit placed on the ski. Minimum, maximum, mean, and range of both ASI and ski edging angle were computed over the gliding phase, giving information about the beginning, end, and throughout the gliding phase. Relative glide time was significantly higher, and minimum and mean ASI were significantly lower in the national- than in the regional-level skiers. Relative glide time was strongly negatively correlated to minimum ASI (i.e., plantar pressure mostly on the foot lateral side at the beginning of gliding phase) and strongly positively correlated to ASI range. These results may reflect a larger body mass transfer above the ski from the beginning of the gliding phase to increase gliding, especially in the national-level skiers. Ski edging angle seems less relevant to discriminate skiers' level of performance. These results have direct consequences on how technique must be taught to young cross-country skiers.

## Introduction

Cross-country skiing skating style comprises different techniques between which skiers change according to speed and slope (Nilsson et al., 2004). Among those technique, V2 (poling phase for every ski thrust, also called G3) has become increasingly used by skiers during competitions, especially in sprint races (Andersson et al., 2010). Several studies examined the cycle characteristics that determine V2 skiing speed and agreed that a strong relationship exists between speed and cycle length (Bilodeau et al., 1992, 1996; Smith, 1992; Boulay et al., 1994; Sandbakk et al., 2011). Elite skiers achieve long cycle lengths with short but strong propulsive phases and long gliding phases (Bilodeau et al., 1992; Stöggl et al., 2008), i.e., low duty cycles. Interestingly, proportional increases of maximal skiing speed, cycle length and absolute gliding phase duration were found in elite skiers during their inter-season preparation (Losnegard et al., 2017). The authors interpreted these changes as improvements in skiers' global fitness and balance, and they associated them to “*possibly reduced friction on snow because of a flatter oriented ski*.” Indeed, the ski flatness to the snow surface (ski edging angle) can influence the gliding phase as it affects ski penetration into snow surface layers (Smith, 2000), explaining practical recommendations from coaches to athletes to reduce friction coefficient by keeping the ski flat during gliding. A recent study showed that peak velocity reached during an incremental test was correlated to a flat or even negative ski edging angle at initial ski–snow contact, i.e., contact with the external edge (Stöggl and Holmberg, 2015). Yet, an older study found that ski edging angle was not significantly related to skiing performance (appraised by cycle velocity and race velocity) during the 50-km Olympic race (Smith and Heagy, 1994). In this study, none of the skiers maintained the ski flat during the gliding phase. In both these studies, ski edging angle was estimated through shank angle relative to vertical, which could bias the results due to possible movements occurring at the ankle in the frontal plane. A measurement taken directly on the ski would improve accuracy, as allowed by modern inertial measurement units (Sakurai et al., 2016).

However, the relevance of studying ski edging angle to improve ski–snow interaction during the gliding phase can be questioned. Ski edging angle only characterizes ski positioning on the snow, which may not be the best variable to assess ski–snow interaction quality and so affecting gliding. Since friction force depends directly on normal force, snow drag during the gliding phase could also be affected by the mediolateral distribution of the load (i.e., skier's body weight) on the ski. Consequently, analyzing mediolateral plantar pressure distribution could be important to better understand the foot–ski–snow interaction during the gliding phase. Indeed, the distribution may provide relevant information about how the skier's mass is transferred above the ski during the gliding phase. Center of pressure patterns during a skating cycle has previously been studied in elite skiers (Smith, 1992; Stöggl et al., 2011). These two studies showed that the center of pressure was approximately centered in the mediolateral dimension during the gliding phase, which implies a homogeneous pressure distribution between the medial and lateral plantar foot area. This homogeneous pressure distribution in the mediolateral dimension would allow skiers to spread the load on the entire foot, and subsequently on the ski, which could reduce snow drag and thus minimize the deceleration during the gliding phase. Even if ski edging has been intuitively associated to a more homogeneous distribution of pressure under the ski, the latter may be better and more directly characterized by mediolateral plantar pressure distribution. Therefore, the main objective of the present study was to determine the effect of the skiers' level of performance on relative glide time and both ski edging angle and plantar pressure mediolateral distribution during on-snow V2 skiing. To further support the association between relative glide time and ski edging angle and plantar pressure mediolateral distribution, the relationships between these variables were assessed using a correlation analysis. It was hypothesized that the higher-level skiers would demonstrate longer relative gliding phases and more homogeneous plantar pressure distributions in the mediolateral dimension rather than flatter ski edging angles.

## Materials and Methods

### Participants

Twelve male cross-country skiers volunteered to participate in the study. All of them reported (i) wearing cross-country skiing boots size 8.5 UK and (ii) having taken part in at least one competition in the two preceding years. They were arranged into a national-level group and a regional-level group based on their score in the ranking system of the French Ski Federation. Selected characteristics of the participants in each group are presented in Table 1. As expected, skiers in the national-level group had a significantly better French Ski Federation Score and a higher weekly cross-country skiing training volume than the regional-level skiers, with respectively very large and large associated effect sizes.

**Table 1:** Selected characteristics (mean ± SD) of the participants in the two groups.

	**National**	**Regional**	***P***	**Effect size**
*N*	6	6	–	–
Age (years)	28.0 ± 6.2	31.3 ± 8.9	0.47	0.47 small
Body mass (kg)	69.2 ± 4.1	70.3 ± 2.3	0.55	0.36 small
Height (cm)	178.3 ± 3.4	180.5 ± 2.7	0.26	0.79 moderate
Pole length (% of height)	89.7 ± 1.0	88.9 ± 1.2	0.26	0.79 moderate
Weekly cross-country skiing volume (h)	14.3 ± 5.9	8.6 ± 2.5	**0.05**	1.38 large
French ski federation score (points)	52.5 ± 35.6	137.8 ± 32.2	**0.01**	2.75 very large

*Bold P-values indicate significant difference between the two groups*.

### Experimental Protocol

Testing was conducted on snow on a freshly groomed, straight skating track. The track was 70 m long with 3 m of positive elevation, resulting in a constant slope of 2.5°. The first 25 m were used for acceleration, and the following 45 m served as measurement area. The participants were instructed to use the V2 skating technique (also called G3, Nilsson et al., 2004), which consists in a symmetrical and synchronous pole push at each leg push off (Bilodeau et al., 1992). Prior to the experiment, a skilled cross-country skier was trained to maintain a 4.2 m s^−1^ speed through the measurement area. During the tests, this skier was placed ahead the participants to fix the speed, and participants were instructed to follow him at about 10 m and to keep this distance constant during the measurements. This speed was chosen to be a representative slow pace training speed (as confirmed by the participants' feedbacks), in order to focus on technical abilities rather than physiological or force production capacities. Actual average speeds over the 45-m measurement area were controlled for each participant (4.4 ± 0.3 m s^−1^, coefficient of variation: 6.5%). Each participant skied the entire track twice: the first trial for habituation to the target speed and the second one for analysis. Testing of each participant was completed within 1 h. The testing of all participants was performed during a period of 6 days during which weather conditions were stable (clear skies). The ski track was prepared every morning by the same operator.

All participants used the same pair of skating shoes (S-Lab Skate Pro, Salomon, Annecy, France), size 8.5 UK, and the same pair of skating skis (S-Lab Equipe 10 Skate, Salomon) mounted with a binding of SNS standard (SNS Pilot Equipe Skate, Salomon). Hygienic insoles were removed from the shoes and replaced by the plantar pressure insoles (PEDAR System, Novel Electronics, Munich, Germany). The participants used their own poles for the tests and carried a small backpack (XA 10+3, Salomon) to transport the acquisition systems (total mass: 1.2 kg). The participants did not report any inconvenience caused by the backpack during skiing. Before each test session, skis were cleaned, prepared with a racing wax (Maplus 40–60 SM Base Solid, Briko, Milan, Italy), and brushed.

### Measurements

Plantar pressures were measured using a Pedar mobile system (Novel GmbH, Munich, Germany). This system sampled at 50 Hz and consisted of two pressure insoles (size 8.5 UK) covered with 99 sensors and a data logger with internal flash memory. The calibration of the insoles was set according to the manufacturer's guidelines before each data recording. As the V2 technique employed is symmetrical, and as ski edging angle was recorded on the right side, only data from the right insole were used for further analysis. The ski edging angle was measured as the ski angle around its longitudinal axis. It was recorded with one Inertial Measurement Unit (MT9, Xsens Technologies B.V., Enschede, Netherlands) solidly fixed with reclosable fastener (3M Dual Lock, 3M, Saint Paul, Minnesota, United States) on the right ski about 2 cm ahead the binding, with the *X* axis aligned with the ski longitudinal axis. The Xsens system sampled at 50 Hz and included a data logger linked by cables to a notebook (Latitude 2100, Dell, Round Rock, USA). Inertial Measurement Units are now widely used in cross-country skiing movement analysis (Myklebust and Nunes, 2011; Marsland et al., 2012; Tjønnås et al., 2019) and were proven to be accurate and reliable in gliding movement analysis (Krüger and Edelmann-Nusser, 2010). The notebook and the Xsens and Pedar acquisition systems were placed in the backpack carried by the participants. The synchronization between both the Pedar and Xsens systems was achieved by striking the right foot and ski on the ground at the beginning of each trial.

### Data Reduction and Analysis

A custom designed mask was applied to the pressure insole data in order to split the total foot area into a medial and a lateral area, resulting in two areas having the same surface (74.7 cm^2^). The average pressure over the total masked foot area was calculated and used to determine cycle characteristics. One step was defined as the period when the ski was in contact with the ground and was composed of a gliding phase followed by a propulsion phase. The gliding phase was defined as in Stöggl et al. (2011); i.e., it started with the placing of the ski on the ground (increase in plantar pressure), leading to a first peak in the total pressure curve, and ended when the total pressure reached a local minimum around midstep. Only data from this gliding phase were used for further analysis.

The relative glide time was expressed relatively to the ski–snow contact duration (T_glide_ in %).

To appraise plantar pressure distribution between the medial and the lateral area, an asymmetry index (ASI, in %) was calculated at each instant of the gliding phase with the following equation (Robinson et al., 1987).

$$\begin{array}{l} \text{ASI}=\frac{{\text{P}}_{\text{medial}}-\;{\text{P}}_{\text{lateral}}}{\left({\text{P}}_{\text{medial}}+\;{\text{P}}_{\text{lateral}}\right)\;}\;\times 100\text{ \%} \end{array}$$

where P_medial_ was the average pressure over the medial area and P_lateral_ was the average pressure over the lateral area. ASI values ranged from −100 to 100%: a negative value indicated a higher pressure on the lateral area, a positive value indicated a higher pressure on the medial area, and an ASI equal to 0 reflected an equally distributed mediolateral pressure. Minimum (ASI_min_), maximum (ASI_max_), range (ASI_range_), and mean (ASI_mean_) ASI were determined during the gliding phase.

The ski edging angle (in °) was the angle around the ski longitudinal axis. A positive angle indicated an internal ski edging, whereas a negative angle indicated external edging, and an angle equal to 0 reflected a ski laid flat on the snow. Minimum (EDG_min_), maximum (EDG_max_), range (EDG_range_), and mean (EDG_mean_) ski edging angle were determined during the gliding phase. Ski edging angle and ASI variables were computed for a maximum of steps in the 45-m measurement area, i.e., four to five skiing cycles.

### Statistical Analysis

Data are presented as mean and standard deviation (SD). Due to the small sample sizes in each group (six participants), differences between the national- and the regional-level group were examined using Mann–Whitney's *U*-tests for all computed variables. Effect sizes were appraised using Cohen's *d* with threshold values of 0.2, 0.6, 1.2, 2.0, and 4.0 for small, moderate, large, very large, and extremely large effects, respectively (Hopkins et al., 2009). After checking distribution normality with the Shapiro–Wilk test, relationships between T_glide_ and the other computed variables were examined using Pearson's product-moment correlations. Correlation coefficients and 95% confidence intervals (CIs) were computed and used to quantify the correlations magnitudes, with values of 0.1, 0.3, 0.5, 0.7, and 0.9 as thresholds for small, moderate, large, very large, and extremely large, respectively (Hopkins et al., 2009). Correlations between ASI variables and the corresponding ski edging angle variables (e.g., ASI_min_ and EDG_min_) were also tested. Statistical significance was set at *P* < 0.05.

## Results

**Figure 1:**
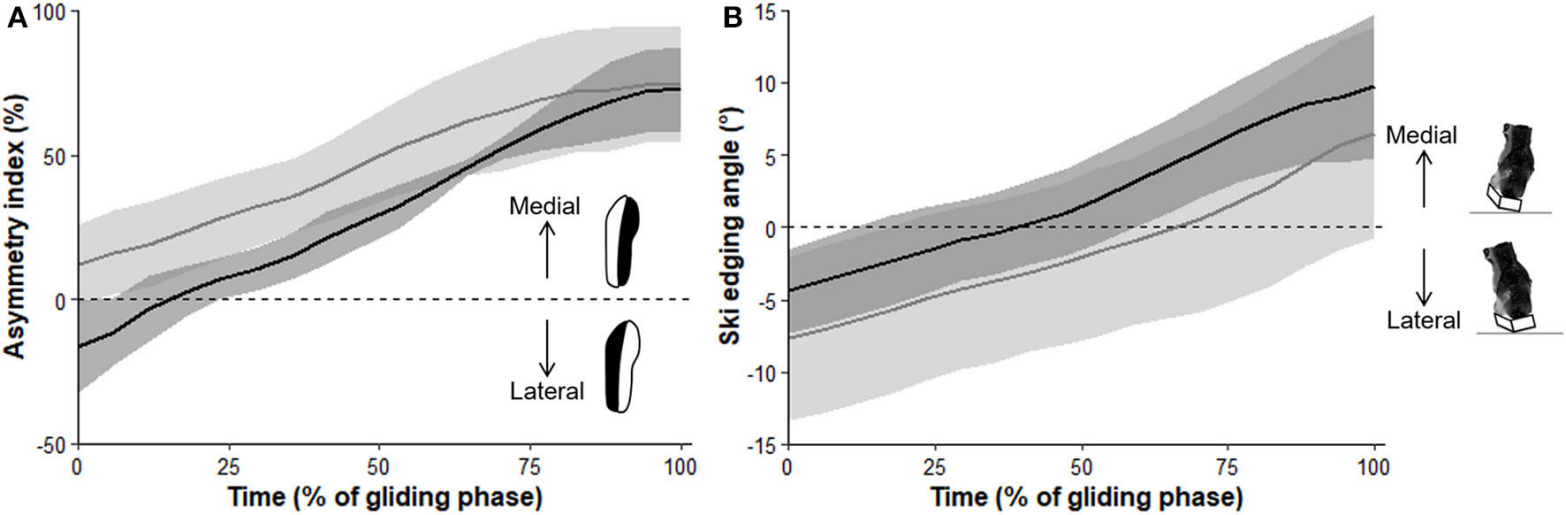
Time courses of the asymmetry index **(A)** and ski edging angle **(B)** during the gliding phase. Data are mean (thick solid lines) ± SD (shaded area) for the national-level group (black) and the regional-level group (gray).

Skiers in the national-level group showed a 15% longer T_glide_ (*P* = 0.04, *d* = 1.71, large effect), a 32.9 %-point lower ASI_min_ (*P* = 0.01, *d* = 2.87, very large effect), and a 14.6 %-point lower ASI_mean_ (*P* = 0.04, *d* = 1.92, large effect) than their regional level counterparts. There was also a trend to a 27.2 %-point higher ASI_range_ for the national-level skiers (*P* = 0.09, *d* = 1.53, large effect). No between-group differences were found for any of the other studied variables (Table 2). These results can be illustrated by the average ASI and ski edging angle signals (Figures 1A,B, respectively), for the two groups. ASI increased quasi-linearly from a minimum to a maximum value during the gliding phase for both groups, with a large between-group difference in ASI_min_ at the beginning of the cycle but similar ASI_max_ values at the end of the gliding phase (Figure 1A). The ski edging angle also increased all along the gliding phase for both groups, but with a similar pattern (Figure 1B).

**Table 2:** Mean ± SD values of the studied variables for the two groups and correlation coefficients (*r*) with the relative glide time.

**Variables**	**Mean** **±** **SD**		**Group effect**	**Effect size**	**Correlations to T**_****glideREL****_		
	**National**	**Regional**	***P***	**Cohen's *d***	***r***	***r* 95% CI**	***P***
T_glide_ (%)	51.8 ± 3.6	43.9 ± 6.2	**0.04**	1.71 large	–	–	–
ASI_min_ (%)	−19.8 ± 10.8	11.6 ± 13.1	**0.01**	2.87 very large	−0.79	[−0.94, -0.39]	** <0.001**
ASI_max_ (%)	74.5 ± 13.8	77.0 ± 15.8	0.82	0.18 trivial	0.32	[−0.31, 0.76]	0.24
ASI_range_ (%)	94.3 ± 20.2	67.1 ± 18.7	0.09	1.53 large	0.79	[0.41, 0.94]	** <0.001**
ASI_mean_ (%)	28.8 ± 2.5	43.4 ± 11.5	**0.04**	1.92 large	−0.31	[−0.75, 0.32]	0.33
EDG_min_ (°)	−6.6 ± 1.8	−7.7 ± 3.3	0.75	0.45 small	0.05	[-0.54, 0.61]	0.88
EDG_max_ (°)	8.9 ± 2.9	6.5 ± 2.8	0.17	0.92 moderate	0.42	[−0.21, 0.80]	0.17
EDG_range_ (°)	15.5 ± 1.6	14.2 ± 2.0	0.34	0.79 moderate	0.60	[0.04, 0.87]	**0.04**
EDG_mean_ (°)	0.3 ± 2.2	−1.6 ± 2.5	0.26	0.88 moderate	0.39	[−0.23, 0.79]	0.21

*Bold P-values indicate statistical significance*.

*ASI/EDG_min_, minimum of the asymmetry index/ski edging angle at the beginning of the gliding phase; ASI/EDG_max_, maximum of the asymmetry index/ski edging angle at the end of the gliding phase; ASI/EDG_range_, range of the asymmetry index/ski edging angle during the gliding phase; ASI/EDG_mean_, mean of the asymmetry index/ski edging angle during the gliding phase*.

**Figure 2:**
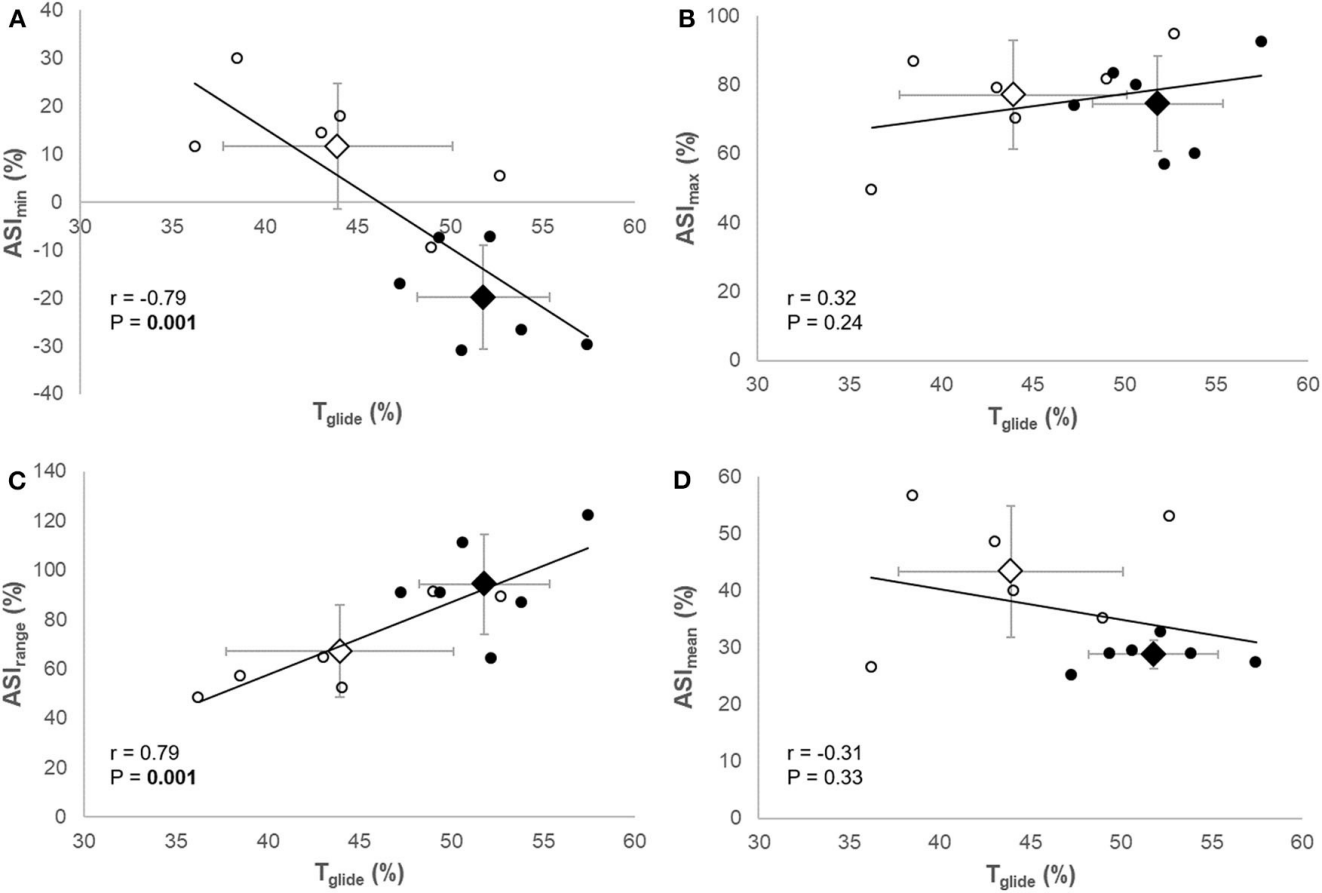
Correlations between the relative glide time (T_glide_) and minimum value [ASI_min_ **(A)**], maximum value [ASI_max_ **(B)**], range [ASI_range_ **(C)**], and mean value [ASI_mean_ **(D)**] of the asymmetry index during the gliding phase. Circles represent individual datapoints and diamonds represent the mean ± SD of the group (open: regional level, solid: national level).

Table 2 shows the correlation coefficients between T_glide_ and ASI and ski edging angle variables. T_glide_ showed a very large negative correlation with ASI_min_ (*r* = −0.79, 95% CI [−0.94, −0.39], *P* < 0.001, Figure 2A) and a very large positive correlation with ASI_range_ (*r* = 0.79, 95% CI [0.41, 0.94], *P* < 0.001, Figure 2C). However, T_glide_ was neither correlated to ASI_max_ (*r* = 0.32, 95% CI [−0.31, 0.76], *P* = 0.24, Figure 2B) nor correlated to ASI_mean_ (*r* = −0.31, 95% CI [−0.75, 0.32], *P* = 0.33, Figure 2D). Only EDG_range_ showed a large positive correlation with T_glide_ (*r* = 0.60, 95% CI [0.04, 0.87], *P* = 0.04), whereas the other ski edging angle variables were not correlated to T_glide_ (Table 2).

Finally, ASI_range_ and EDG_range_ showed a large correlation (*r* = 0.58, 95% CI [0.01, 0.87], *P* = 0.05), while no other correlations were found between the other ASI and ski edging angle variables: *r* = −0.02 (CI [−0.59, 0.56]) for ASI_min_ vs. EDG_min_ (*P* = 0.95), *r* = −0.27 (CI [−0.73, 0.36]) for ASI_max_ vs. EDG_max_ (*P* = 0.38), and *r* = −0.12 (CI [−0.65, 0.49]) for ASI_mean_ vs. EDG_range_ (*P* = 0.70).

## Discussion

The purposes of this study were to assess the differences in relative glide time and both ski edging angle and plantar pressure mediolateral distribution in skiers of different levels and to further investigate the relationships between these variables. It was hypothesized that the higher-level skiers would demonstrate longer relative gliding phases and more homogeneous plantar pressure distributions in the mediolateral dimension rather than flatter ski edging angles.

First, T_glide_ was significantly greater in skiers of the national- compared to the regional-level group. These results confirm the gliding phase relative duration as a determining factor of ski skating performance (Bilodeau et al., 1992; Stöggl et al., 2008; Losnegard et al., 2017). It should be emphasized that T_glide_ is a computation of *relative* glide time. Interestingly, *absolute* glide time was computed as an additional analysis and was not different between groups (national: 0.61 ± 0.08 s vs. regional: 0.56 ± 0.08 s, *P* = 0.38). This means that the duration of the gliding phase itself is not what differentiates between skiers' level, but rather the ability to lengthen the gliding phase for a given cycle time. This ability could be attributed to both a more effective push-off and/or more effective gliding phase. For that matter, both ASI_min_ and ASI_mean_ were significantly lower, and ASI_range_ tended to be larger in skiers of the national- compared to the regional-level group, which gives some credit to the more effective gliding phase interpretation. Figure 1A shows that the national-level skiers began the gliding phase with a plantar pressure more distributed toward the lateral side whereas the regional-level skiers had a plantar pressure already mostly distributed in the medial side. At the end of the gliding phase, the skiers of both groups had approximately the same plantar pressure distribution, with most of the pressure on the medial area that is needed to optimize the following push-off (Smith, 2000). The national level skiers seem to be able to position their center of mass more laterally relatively to the ski than the lower-level skiers in preparation to the gliding phase. This may allow them to put their body mass more directly above the ski (Smith and Heagy, 1994; Stöggl and Holmberg, 2015), potentially giving them more stability (with less muscle isometric contraction) to be able to lengthen the gliding phase. Further research including propulsion forces analysis is needed to understand whether the longer gliding phases demonstrated by the higher-level skiers can also be attributed to more effective push-off. Oppositely, no differences were observed between the national and regional level skiers for any of the ski edging angle variables (Figure 1B). Thus, ski edging angle may be of lesser importance in discriminating the skiers' level of performance.

Moreover, T_glide_ was positively correlated to ASI_min_ and ASI_range_ (Figures 2A,C, respectively), which supports the association between differences in T_glide_ and differences in ASI variables observed between groups. These results indicate that skiers with the longest gliding phases had a greater ASI variation during gliding because of a lower value at the beginning of the step, i.e., more pressure on the foot lateral area. No correlation existed between T_glide_ and ASI_max_ (Figure 2B), indicating that the pressure distribution at the end of the gliding phase did not influence the gliding phase duration. As previously mentioned, this evolution of the plantar pressure distribution from the lateral to the medial area might be associated with a mass transfer above the ski during the gliding phase. This interpretation is in line with the results of a recent study showing that the majority of the V2 kinematics variance was occurring in the mediolateral dimension, and especially for the center of mass kinematics (Gløersen et al., 2018). Indeed, forces (and consequently pressure) applied to the foot are important to maintain stability on the ski, particularly during the gliding phase. The plantar pressure distribution can thus inform indirectly on the position of the skier's center of mass above the ski. The present results suggest that longer gliding phases were allowed by the ability to position the center of mass directly above the ski (and even laterally) at the beginning of the gliding phase, as also proposed by Stöggl and Holmberg (2015). These results are also in line with those of Losnegard et al. (2017) who observed that longer glide times were associated with a greater center of mass mediolateral movement amplitude during the skiing cycle. Further research is needed to confirm the relationships between relative glide time, plantar pressure mediolateral distribution, and center of mass mediolateral movements using motion capture measurements.

We also hypothesized that a flat ski edging angle during the gliding phase would not necessarily be associated to relative glide time. This second hypothesis was partially confirmed since a significant correlation was found between T_glide_ and EDG_range_. However, neither EDG_range_ nor any other ski edging angle variable was different between the skiers of national and regional level. Thus, it can be argued that ski edging angle variables could be of less importance in quantify skiers' technical ability. Indeed, all skiers did have ski edging angle variations during the gliding phase, consistently with the observations of Smith and Heagy (1994). The present results also showed that neither ski edging angle at ski–snow contact (EDG_min_) nor average ski edging angle during the gliding phase (EDG_mean_) was related to relative glide time or performance. Thus, the widespread practical recommendation from coaches to athletes of keeping the ski flat on the snow might not be relevant.

However, even if there was no period with ~0° edging angle during the gliding phase for any skier, the minimum and maximum ski edging angles were only ± 7–8° from flat on average (Table 2). It seems that all participants were able to hold this modest amount of edging, which may have had little effect on ski glide (Smith, 2000). One limitation is that ski edging angle was measured in a global reference frame (i.e., earth based); thus, absolute values could have been slightly biased by the snow track inclination. As the track inclination in the present study was only 2.5°, the maximal potential bias was assumed to be very low and not to challenge the present findings.

It is worth mentioning that apart from a correlation between ASI and ski edging angle range, none of the other ASI and ski edging angle variables were correlated to each other. This shows that plantar pressure distribution and ski edging angle bring different information about a skier's technique. This result might seem a little surprising as rotation of the ski along its longitudinal axis (i.e., edging) can be initiated by shifting the skier's mass on one edge or the other. However, small movements in the frontal plane may have occurred at the ankle, thus impacting edging angle but not plantar pressure distribution. This is supported by previous studies on postural control on inclined surfaces, which showed low shift of center of pressure position when standing on an inclined surface (Kluzik et al., 2005; Lin and Nussbaum, 2012). This confirms that longer gliding phases in the present study may have been achieved by a better mediolateral mass transfer above the ski while keeping a moderate edging angle.

One limitation of the present study is that only specific factors (i.e., ski edging and plantar pressure distribution) influencing glide time were studied. Further research is needed to get a more extensive understanding of which factors influence ski edging and plantar pressure distribution during the gliding phase in V2 skating. Since upper body strength was previously demonstrated as a strong predictor of V2 skating performance (Stöggl et al., 2011), pole forces might be interesting to measure to know whether they affect glide time directly through higher propulsive power or indirectly through improving mediolateral balance and in turn plantar pressure distribution during gliding phase. Another limitation concerns the relatively low speed used in this study (4.2 m s^−1^). This speed was chosen to be a representative slow training pace, and this was confirmed by the participants' feedback. This allowed the skiers of both groups to ski at a low physiological intensity and produce relatively moderate forces, so that the results were assumed not to be due to differences in physiological of force production capacities between the skiers. In other words, this slow speed permitted to rule out a potential bias, and the present results can be quite certainly attributed to different technical abilities between the skiers. A follow-up of this research should include higher speeds and test the influence of skiing velocity on the ski edging and plantar pressure distribution variables. Given the results obtained presently at low speed, it can be reasonably assumed that similar observations would be made at higher velocities. Finally, the concurrent use of group comparisons and correlation analyses is delicate as the groups may directly be responsible for the correlations if they form two distinct clusters. The present results suggest it was not the case here since the data points rather scatters as a continuum, including within the groups (Figure 2). Yet, given the low number of participants, further research is mandatory more participants of homogeneous level to further support the relationships observed between relative glide time and ASI variables.

The present results may have implications for V2 technique teaching to young cross-country skiers. They support that for a given speed, an increased relative gliding time is related to performance, giving the skier a longer recovery between two push-offs. While a traditional advice from coaches and instructors is to focus on keeping the ski flat to enhance gliding, the present results also imply that the skiers must try to put a substantial part of their mass on the lateral foot area at the beginning of the step, in order to have a large transfer to the medial area during the gliding phase. Further research is needed to assess the relationship between gliding time and push-off effectiveness, in order to complement the aforementioned implications for coaching. Future work should also study the variables measured in the present study at higher speeds and in different ski skating techniques.

## Data Availability Statement

The raw data supporting the conclusions of this article will be made available by the authors, without undue reservation.

## Ethics Statement

The studies involving human participants were reviewed and approved by the local institutional ethics committee of the Savoie Mont-Blanc University. The patients/participants provided their written informed consent to participate in this study.

## Author Contributions

SP set up and conducted the experiment, processed the data, and wrote the manuscript. FH participated in the experiment setup and reviewed the manuscript. GM participated in the interpretation of the results and reviewed the manuscript. NH reviewed the manuscript. PS set up the experiment, participated in the interpretation of the results, and reviewed the manuscript. All authors contributed to the article and approved the submitted version.

## Conflict of Interest

The authors declare that the research was conducted in the absence of any commercial or financial relationships that could be construed as a potential conflict of interest.
